# Atomic-level structural and chemical analysis of Cr-doped Bi_2_Se_3_ thin films

**DOI:** 10.1038/srep26549

**Published:** 2016-05-25

**Authors:** A. Ghasemi, D. Kepaptsoglou, L. J. Collins-McIntyre, Q. Ramasse, T. Hesjedal, V. K. Lazarov

**Affiliations:** 1 Department of Physics, University of York, York YO10 5DD, United Kingdom; 2 SuperSTEM Laboratory, SciTech Daresbury Campus, Daresbury WA4 4AD, United Kingdom; 3 Department of Physics, Clarendon Laboratory, University of Oxford, Oxford OX1 3PU, United Kingdom

## Abstract

We present a study of the structure and chemical composition of the Cr-doped 3D topological insulator Bi_2_Se_3_. Single-crystalline thin films were grown by molecular beam epitaxy on Al_2_O_3_ (0001), and their structural and chemical properties determined on an atomic level by aberration-corrected scanning transmission electron microscopy and electron energy loss spectroscopy. A regular quintuple layer stacking of the Bi_2_Se_3_ film is found, with the exception of the first several atomic layers in the initial growth. The spectroscopy data gives direct evidence that Cr is preferentially substituting for Bi in the Bi_2_Se_3_ host. We also show that Cr has a tendency to segregate at internal grain boundaries of the Bi_2_Se_3_ film.

Three-dimensional topological insulators (3D-TIs) are insulating in their bulk, but possess a surface state that arises from the topologically non-trivial bulk band structure which is ‘robust’ as it is protected by time-reversal symmetry (TRS)^1^,^2^,^3^,^4^. Strong spin-orbit coupling is commonly the origin of the nontrivial topology of the bandstructure. Among the various Bi-based TIs that have been studied, Bi_2_Se_3_ is one of the most promising candidates for future device applications. Crystalline Bi_2_Se_3_ has a rhombohedral structure and its unit cell is made up of three weakly bonded quintuple layers (QLs)^4^. It has a single Dirac cone in the Brillouin zone and a nontrivial (bulk) band gap of 0.3 eV^5^. Upon doping with magnetic dopants, TRS may be broken and Dirac electrons may become effectively massive^6^. The combination of magnetism with TIs can lead to a number of interesting phenomena, such as the point charge-induced magnetic monopole and topological contributions to the Faraday and Kerr magneto-optical effects^7^. According to first-principle calculations, doping TIs with V, Cr, Mn or Fe can break TRS and open a band gap in the surface bandstructure^8^. Long-range ferromagnetic order has been reported in materials such as Cr- and Sm-doped Bi_2_Se_3_^7^,^9^,^10^,^11^, Mn-and Fe-doped Bi_2_Se_3_^12^,^13^, and Bi_2_Te_3_^14^,^15^, as well as V-, Cr- and Mn-doped Sb_2_Te_3_^7^,^16^. The latter system has been used to demonstrate exotic quantum phenomena such as the magnetoelectric and the quantum anomalous Hall effect (QAHE)^17^. Most importantly, the interaction between topologically protected surface states and ferromagnetism is expected to give rise to different unconventional spintronic effects for device applications.

The location of the dopants in the Bi chalcogenide matrix depends highly on the type of dopant, e.g., its ionic radius and ability to form undesired chalcogenide compounds, its concentration and the growth conditions. In Cr:Bi_2_Se_3_, dopants can either substitute for Bi or incorporate between the QLs in the van der Waals gap^18^,^19^. First principle calculations predict that the Bi sites are the most energetically favorable substitutional sites for Cr^4^,^8^. Formation energies of Cr being in the van der Waals gap are 0.29 eV higher compared to the substitutional scenario, and interstitial Cr within the QLs is much less favorable^4^.

In this letter we show that Cr can be incorporated in the Bi_2_Se_3_ structure with no phase segregation. As predicted by calculations, we found that Cr is incorporated substitutionally on Bi sites. In addition we demonstrate that Cr can segregate on the grain boundaries which are inevitably present in thin film growth. This segregation of Cr is correlated to the grain boundary density. Their effect on the surface state of Bi_2_Se_3_ would depend on the geometry and the spatial extension of the boundary defects. By controlling the defect density, the amount of Cr that segregates can be minimized, hence a homogeneous Cr distribution could in principle be achieved even at higher Cr-dopant concentrations.

In order to determine the location and distribution of Cr dopands in the film we have performed extensive state of the art high-angle annular dark field (HAADF) imaging and atomically resolved Electron Energy loss spectroscopy measurements (EELS) in an aberration corrected Scanning Transition Electron Microscope (STEM). Figure 1a shows a low magnification cross-sectional view of the molecular beam epitaxy (MBE)-grown Cr:Bi_2_Se_3_ thin film on *c*-plane Al_2_O_3_. The atomic number dependence (~*Z*^2^) HAADF-STEM images clearly outlines the Bi_2_Se_3_ film (bright contrast) and the Al_2_O_3_ substrate (darker region). The growth of the Cr:Bi_2_Se_3_ film (thickness ~100 nm) is mediated by granular growth driven by screw dislocations (Supplementary Fig. S2). This growth process results in a film of uniform thickness with a smooth surface morphology as indicated also by the Reflection high-energy electron diffraction (RHEED) patterns taken at the end of the film growth (Supplementary Fig. S1). The selected area electron diffraction (SAED) pattern (Fig. 1b) obtained from the substrate-film interfacial region shows that the Cr:Bi_2_Se_3_ is single-crystalline and that it is grown epitaxially on Al_2_O_3_ (0001) with the following crystallographic relationships: 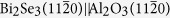 and Bi_2_Se_3_ (0001) ‖ Al_2_O_3_ (0001).

The structural ordering of the film is shown in HAADF images acquired along the 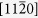 zone axis (Fig. 2). The abrupt change in the HAADF image contrast indicates a chemically abrupt substrate-film interface, and the white fringes are showing the continuous QL structure of the film. Figure 2b gives a closer view of the interfacial region between the Al_2_O_3_ and Cr:Bi_2_Se_3_. The image shows, that while interface with the Al_2_O_3_ is chemically abrupt - the film is not fully ordered at the initial stage of the growth. In fact, it requires about 3–4 atomic layers before the regular QL growth of Bi_2_Se_3_ is realized. Once the QL growth is established, the subsequent film is continuously ordered as confirmed by x-ray diffraction (XRD) and the atomic force microcopy (AFM) imaging (Supplementary Figs S2 and S3); along this crystallographic orientation Bi and Se atomic columns do not overlap, hence Bi and Se atomic columns are easily distinguishable due to the much higher atomic number of Bi compared to Se. The inset in Fig. 2b is a high-magnification HAADF image of a QL clearly showing the atomic stacking of the Bi and Se atomic columns.

Next we focus on determining the location of the Cr dopants in the Bi_2_Se_3_ matrix. Direct imaging of Cr by HAADF is not feasible due to the low *Z* of Cr (Z_Cr_ = 24) in comparison to Bi and Se and the low concentration of Cr in the film. In order to get an unambiguous signature of the Cr present in the film we performed spatially resolved electron energy loss spectroscopy (EELS) mapping, by rastering the electron probe serially across a defined region and collecting an EEL spectrum at each point. Chemical maps where then created by integrating at each point of these spectrum images the spectrum intensity over a ∼20 eV window above the EELS edge onsets, while HAADF intensity signal was simultaneously acquired allowing for unambiguous correlation of the chemical information to the structural image.

Figure 3a shows a HAADF-STEM survey image in which the region selected for EELS measurements of the Cr *L*_2,3_ edge is highlighted (Fig. 3b). The map of the Cr *L*_2,3_ signal intensity is shown in Fig. 3c (data treated by Principle Component Analysis (PCA)). Similar to previous reports on the same system^10^, the map shows a uniform Cr distribution throughout the film without any signs of Cr diffusion into the substrate, or Cr segregation at the interface or surface of the sample (Supplementary Fig. 5). In order to determine the position of Cr dopants in the Bi_2_Se_3_ structure we performed atomically resolved EELS measurements of the bulk are of the film. Figure 3e shows an atomically resolved Cr *L*_2,3_ EELS map (data treated by PCA, for more details and raw data see the Fig. S4 in the supplementary section). Direct comparison of the Cr chemical map intensities (see plotted integrated intensities in Fig. 3f) with the simultaneously acquired HAADF signal (Fig. 3d), shows that Cr is in registry with Bi columns, i.e., Cr is substituting for Bi, a direct confirmation of the first principles calculations^4^,^7^. This is further highlighted by atomically-resolved EELS measurements including both the Cr *L*_2,3_ and Se *L*_2,3_ signals (Fig. S6) showing clearly the anti-correlation between the Cr and Se signals. It should be noted that the Cr elemental maps are displayed as normalised intensities (stretching the image contrast, for visual convenience). While these maps are indicative of the relative spatial distribution of the elements, they should therefore not be interpreted as a quantification of the sample composition.

In addition to the Cr substitution of Bi atoms, we have also found that Cr segregates at the grain boundaries which are rather common in MBE-grown Bi_2_(Se,Te)_3_ films due to the van der Waals epitaxy and substrate surface steps^20^,^21^,^22^. Figure 4 shows an elemental map of Cr in the region where grain boundaries are present. In the survey scan (Fig. 4a), the area including the boundary is indicated. Comparing the HAADF STEM image (Fig. 4b) simultaneously acquired with the EELS signal, to the Cr *L*_2,3_ intensity map, a clear increase of the Cr *L*_2,3_ signal is seen at the grain boundary (dark contrast in Fig. 4a,b) compared to regular QL regions (Fig. 4c,e), data treated by PCA, for more details and raw data see the Figs S5 and S6 in the supplementary section). This implies that grain boundaries act as ‘sinks’ for Cr dopants. It is to be expected that segregation of Cr at the boundaries can be significant when Cr concentrations in the films are large. On the other hand, the corresponding Se map of the same region shows a uniform distribution throughout the sample (Fig. 4d). In order to access whether the Cr segregated at the grain boundaries has a different chemical state than the fully substitutional Cr in the film, we turn to the near edge fine structure of the Cr *L*_2,3_ EELS signal. More specifically we access the ratio between the *L*_2_ and *L*_3_ peaks of the Cr core loss edge, a method commonly used to access the valence state of transition metals. For this the background subtracted Cr *L*_2,3_ edge of the areas highlighted in Fig. 4b are normalized to the maximum of intensity of the L_3_ peak (Supplementary Fig. S7). It can be seen that *L*_3_/*L*_2_ intensity ratio does not appear to change between the bulk and of grain boundary regions indicating that the nominal valence state of Cr is the similar throughout the specimen (Supplementary Fig. S7)^23^,^24^.

The uniform distribution of Cr in the film agrees well with the measured ferromagnetic properties^22^, in contrast to a recent report of Cr segregation at the surface resulting in superparamagnetic behavior of Cr:Bi_2_Se_3_. Even though the segregation of the Cr is not desired in the films, as long as the majority of the grain boundaries are within the interior of the film (as observed in this study) they should not strongly affect the Dirac surface states, which are macroscopic states spanning over the entire surface of the contiguous film. In order to suppress the formation of grain boundaries, we suggest the growth at lower growth temperatures and lower growth rates.

In summary, we presented a structural study of a single-crystalline, Cr-doped Bi_2_Se_3_ film on Al_2_O_3_ (0001). Structural and spectroscopic studies using aberration-corrected STEM-EELS have shown that Cr incorporation in Bi_2_Se_3_ film proceeds via substitution of Bi atoms. For the investigated Cr concentration of 4.6 at-% the dopant does not disturb the rhombohedral Bi_2_Se_3_ structure. Additionally, we find a segregation of Cr at the grain boundaries of the films. By controlling the density of defects and controlling the growth conditions Cr segregation at the boundaries can be minimized and incorporation of Cr in Bi_2_Se_3_ films can be achieved via uniform substitution of Bi.

## Methods

The Cr:Bi_2_Se_3_ thin film samples were prepared by MBE on *c*-plane sapphire substrates, following the recipe described in ref. 9. The MBE growth chamber has a base pressure of 1 × 10^−10^ Torr. RHEED was used to monitor the growth *in-situ*, and the streaky patterns (Supplementary Fig. S1) are indicative of 2D growth. The AFM image (Supplementary Fig. S2) illustrates the spiral islands, common for *c*-axis oriented Bi_2_Se_3_ films, with QL-high steps (~1 nm). The islands (or grains) have a lateral dimension of typically ~150 nm, and are separated by trenches (grain boundaries). The sample used in this study has a thickness of 103 nm, as determined by x-ray reflectivity (XRR) and RBS. The sample composition was determined by RBS to be 4.6 at-% Cr, 35.3 at-% Bi, and 60.1 at-% Se, bringing the (Cr + Bi):Se ratio to 2:3, indicative of Cr being substitutional on Bi sites^9^. The XRD pattern (Supplementary Fig. S3) shows the (00*l*) family of peaks representative of Bi_2_Se_3_, and the extracted *c*-axis lattice parameter of ~28.65 Å is slightly larger than the literature value for Bi_2_Se_3_ (ICSD 617072). The magnetic saturation moment of the sample is ~2.1 μB/Cr and the Curie temperature 8.5 K^9^.

Cross-sectional transmission electron microscopy (TEM) specimen preparation was carried out by focused ion beam (FIB) methods using a FEI Nova 200 NanoLab high-resolution field emission gun scanning electron microscope (FEGSEM). A layer of Pt was deposited to protect the film from Ga ion implantation and damage.

Structural characterization has been performed by Transmission Electron Microscopy. The SAED were obtained using a JEOL 2000 EX. STEM imaging and EELS measurements were performed in a Nion UltraSTEM100^TM^ equipped with a Gatan Enfina spectrometer. The microscope was operated at 100 kV, with a convergence angle of 30 mrad; at these optical conditions the electron probe size is determined to be 0.9 Å. The inner detector angle for HAADF-STEM imaging was 76 mrad. The native energy spread of the electron beam for EELS measurements was 0.3 eV; with the spectrometer dispersion set at 03 eV/channel & 1 eV/channel, yielding effective an energy resolution of 0.9 eV and 3 eV, respectively. The EELS collection angle was 33 mrad. For enhancing the contrast of the atomically-resolved spectra, a noise-reduction routine was applied using principal component analysis (CiMe^−^ plugin for Gatan’s Digital Micrograph 2.3 software suite^25^). Following PCA, chemical maps were created by integrating at each point of these spectrum images the spectrum intensity over a ∼20 eV window above the Cr *L*_2,3_ and Se *L*_2,3_, EELS edge onsets after background subtraction using a power law model.

### Data Availability

All data created during this research are available by request from the University of York Data Catalogue https://dx.doi.org/10.15124/e3abd365-2cc0-4938-9bc1-24ee4b4db6b1.

## Additional Information

**How to cite this article**: Ghasemi, A. *et al.* Atomic-level structural and chemical analysis of Cr-doped Bi_2_Se_3_ thin films. *Sci. Rep.* **6**, 26549; doi: 10.1038/srep26549 (2016).

## Supplementary Material

Supplementary Information

## Figures and Tables

**Figure 1:**
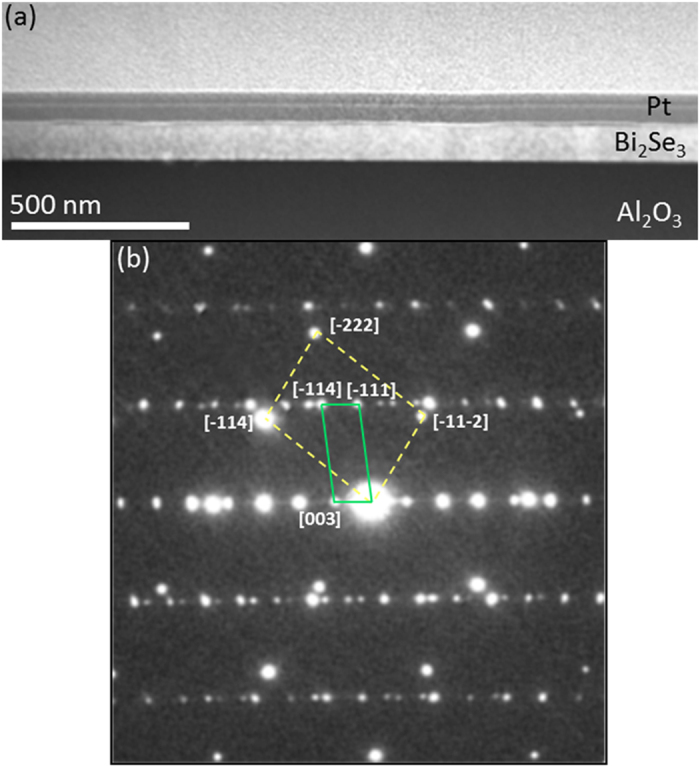
(**a**) Low magnification HAADF-STEM image of the Bi_2_Se_3_ film on Al_2_O_3_ (0001). (**b**) SAED from the Bi_2_Se_3_/Al_2_O_3_ interface region along the 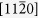 crystallographic direction. The dashed (yellow) and solid (green) rectangles are showing the projected unit cells of Al_2_O_3_ and Bi_2_Se_3_, respectively.

**Figure 2:**
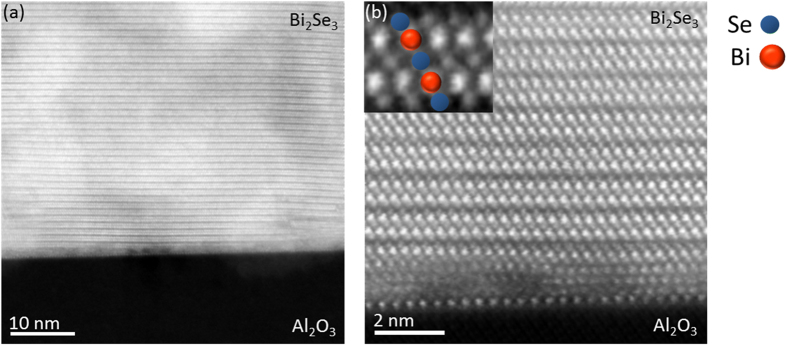
(**a**) HAADF-STEM overview image from the interfacial region between the film and substrate. (**b**) Magnified image of the film at the interface. A smooth interface and regular Bi_2_Se_3_ quintuple layers are found, except for the first layer which appears to be highly disordered. The inset shows magnified image of a quintuple layer with overlaid structural model; Bi (red) and Se (green) atomic columns.

**Figure 3:**
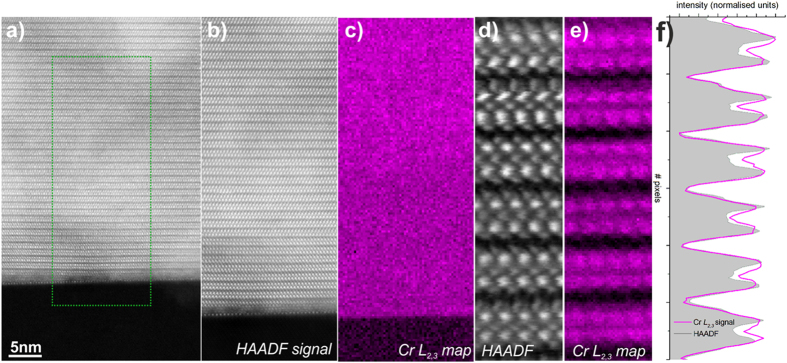
Elemental mapping of 4.6% Cr doped Bi_2_Se_3_. (**a**) HAADF-STEM survey image from Bi_2_Se_3_ film and Al_2_O_3_ substrate. (**b**) HAADF-STEM signal from the region of interest outlined in (**a**) produced simultaneously with the EELS acquisition. (**c**) Cr *L*_2,3_ EELS signal showing uniform distribution of Cr throughout the quintuple layers contained within the region of interest. (**d**) The atomically resolved HAADF-STEM image from the film area recorded simultaneously with the EELS signal shown in (**e**). (**e**) Spatially resolved intensity of Cr *L*_2,3_ edge signal, showing that Cr is substituting Bi in the quintuple layers shown in (**d**). (**f**) intensity profiles of Bi atomic columns and Cr elemental map showing the direct spatial correlation between these two signals.

**Figure 4:**
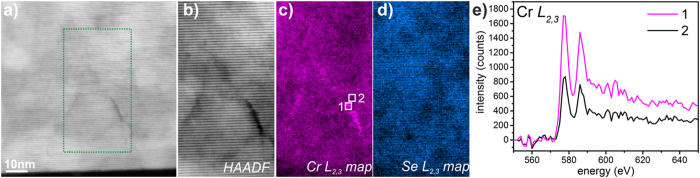
Grain boundary imaging and elemental analysis. (**a**) HAADF-STEM survey image. (**b**) HAADF-STEM signal from the region of interest outlined in (**a**) produced simultaneously with the EELS acquisition. (**c**) Cr *L*_2,3_ EELS signal showing Cr segregation along the grain boundaries. (**d**) Se *L*_2,3_ EELS signal showing uniform distribution of Se in the film. (**e**) background subtracted Cr *L*_2,3_ edges obtained from the grain boundary and off boundary regions labeled as number 1 and 2 in (**c**) showing enhanced Cr signal at the grain boundary.
