# Supplementary material for: Atomic-level structural and chemical analysis of Cr-doped Bi2Se3 thin films
Source: Sci Rep. 2016 May 25;6:26549. doi: 10.1038/srep26549 (PMC4879552; doi:10.1038/srep26549)
Supplement: Supplementary Information [file srep26549-s1.doc]

Supplementary Information for

**Atomic-level structural and chemical analysis**

**of Cr-doped Bi_2_Se_3_ thin films**

A. Ghasemi,^1^ D. Kepaptsoglou,^2^ L. J. Collins-McIntyre,^3^ Q. Ramasse,^2^

T. Hesjedal,^3^ and V. K. Lazarov^1^

^1^Department of Physics, University of York, York YO10 5DD, UK

^2^SuperSTEM Laboratory, SciTech Daresbury Campus, Daresbury WA4 4AD, UK

^3^Department of Physics, Clarendon Laboratory, University of Oxford, Oxford OX1 3PU, UK

**Here, additional information about the sample under investigation are given. First, we show the reflection high-energy electron diffraction images obtained *in-situ*, followed by atomic force micrographs, obtained *ex-situ*, and X-ray diffraction. Details of the PCA of EELS spectra are provided, as well as discussion of the valence state of the Cr from the EELS data.**

1. **Reflection high-energy electron diffraction**

**Figure S1** presents the reflection high-energy electron diffraction (RHEED) patterns obtained *in-situ* at the end of the thin film growth in two azimuthal directions. The RHEED images are characterized by sharp, well-defined streaks, which show a 3-fold symmetry which is visible upon rotation. The spacing of the streaks in the two directions, which have an angular separation of 30°, differs by a factor of$\sqrt{3}$, as expected from the *R$\bar{3}$m* space group.


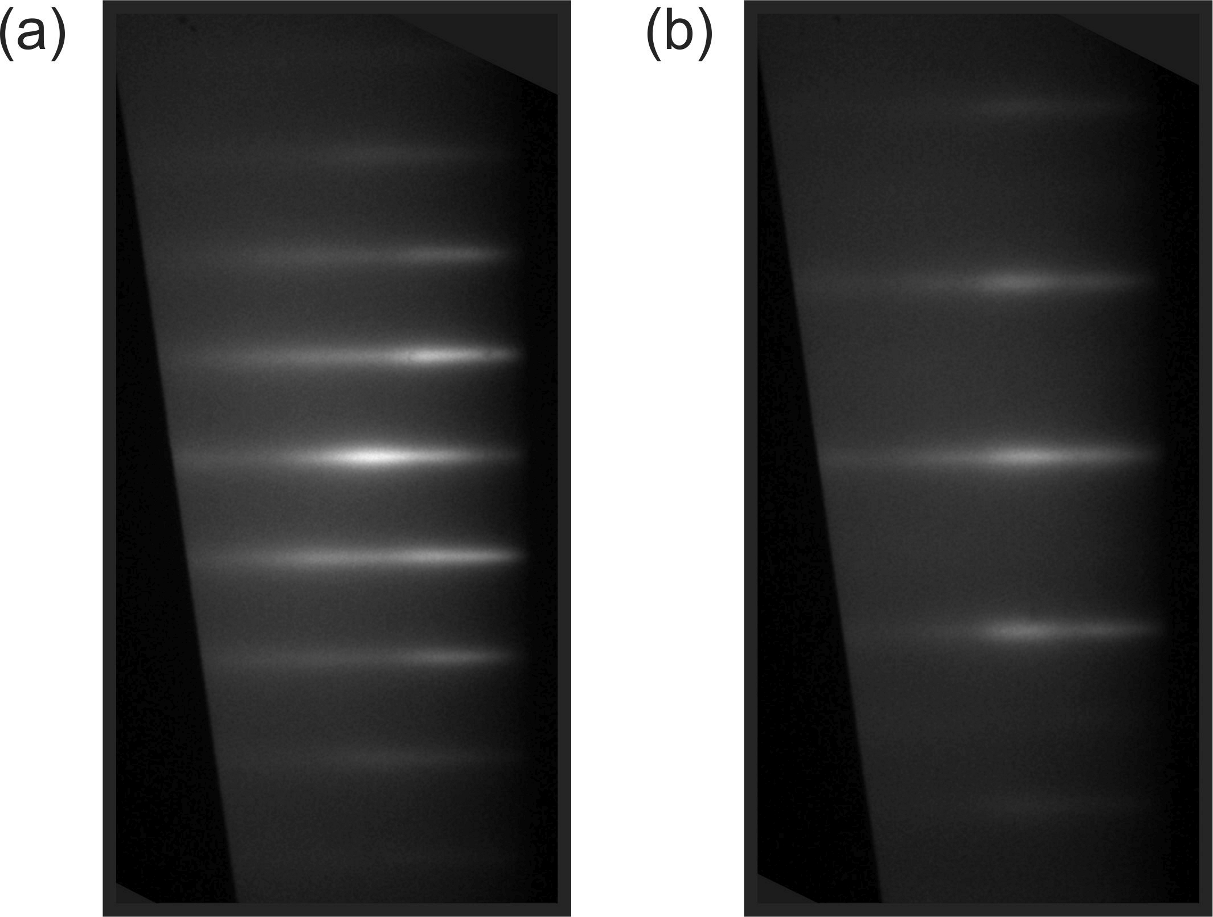


**Figure S1**: RHEED images of the Cr-doped Bi_2_Se_3_ film recorded in-situ at the end of the film growth along the (a) [10$\bar{1}$0] and (b) [11$\bar{2}$0] azimuths, respectively.

1. **Atomic force microscopy (AFM)**

The AFM images shown in **Figure S2** were acquired using a Veeco Multimode V AFM in tapping mode. AFM scans are useful for understanding the growth mode and morphology of the films.


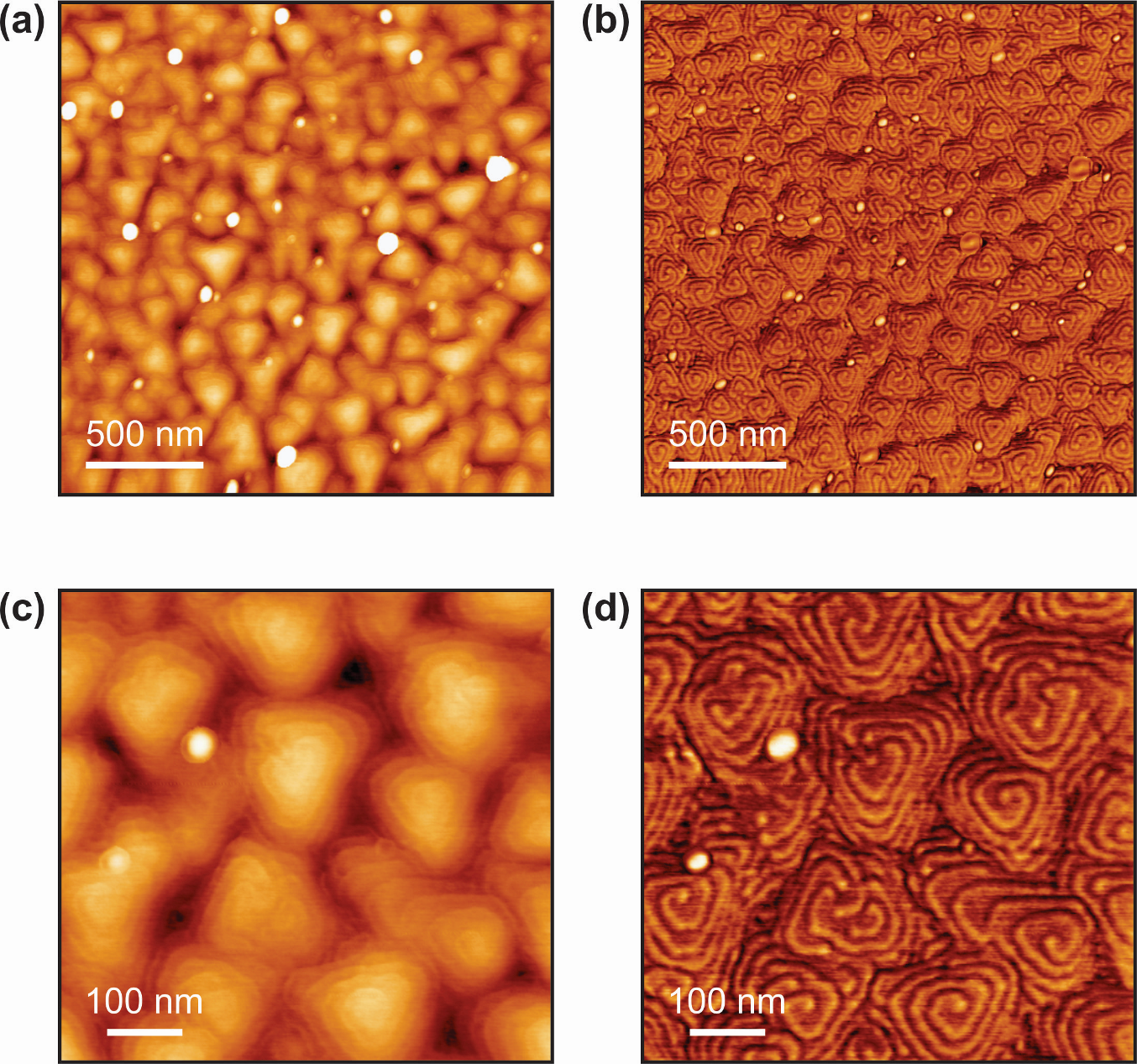


**Figure S2**: Tapping mode AFM images showing the height in the left column (a,c) and the phase in the right column (b,d). The overview scans (a,b) illustrate the dominance of the triangular growth islands, separated by trenches. The bright spots are due to surface contamination as a result of sample cleaving. The zoomed-in images in (c,d) show an average triangular island size of ~150 nm. The ~1-nm-high quintuple layer steps of the terraces are clearly resolved. In the phase image the spiral growth mode is visible.

**3. X-ray diffraction (XRD)**

The XRD and rocking curve measurements were carried out on a Bruker D8 x-ray diffractometer with a Cu anode (λ = 1.54 Å). Incident optics were set with a Ge (220) 2-bounce monochromator, 2.5° Soller slits and 1 mm beam mask. The receiving optics used 2.5° Soller slits arriving at either an area detector (XRD) or a scintillator counter (rocking curves). **Figure S3** shows a 2Θ-ω scan of the Cr-doped film. The film only exhibits the (00*l*) family of peaks, as labeled in the figure. The sapphire peaks are indicated as well. In general, despite the comparably high doping concentration of *x =* 0.12 [in (Cr_x_Bi_1-x_)_2_Se_3_], the crystalline quality of the films as evidenced by the peak width remains very good, in contrast to the work reported in Ref. S1.


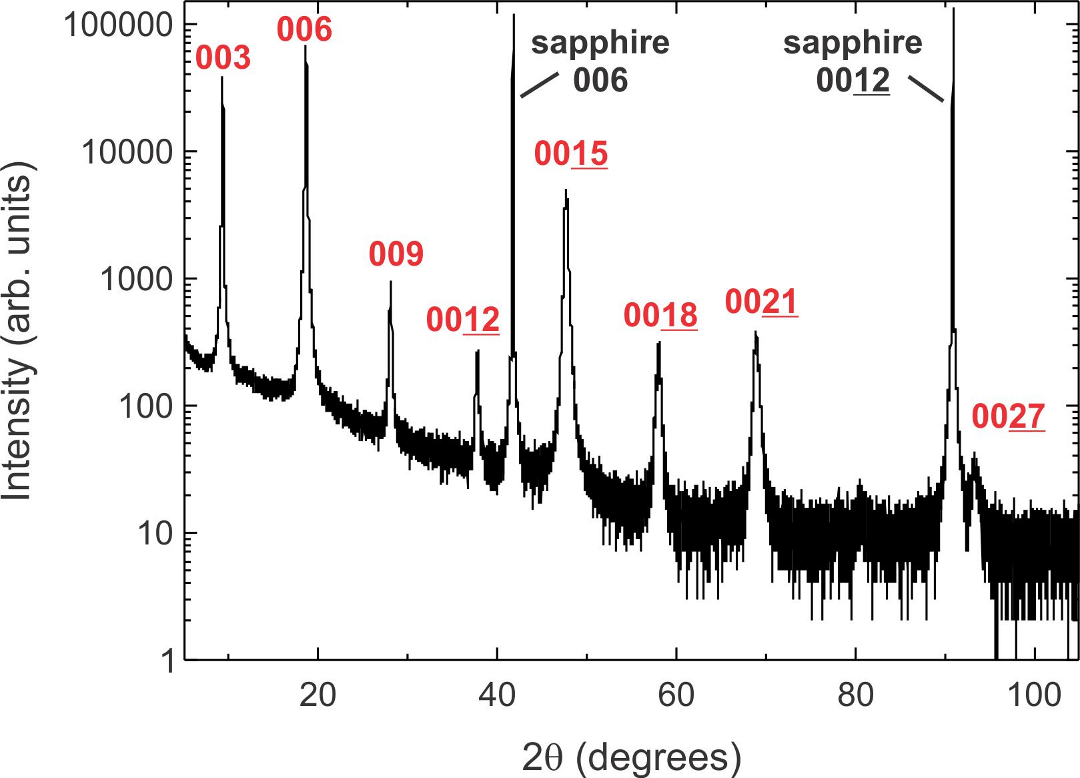


**Figure S3**: X-ray diffraction of the Cr-doped thin film using for this investigation. The film peaks are labeled in red and the sapphire substrate peaks in black.

**Electron Energy Loss Spectroscopy**


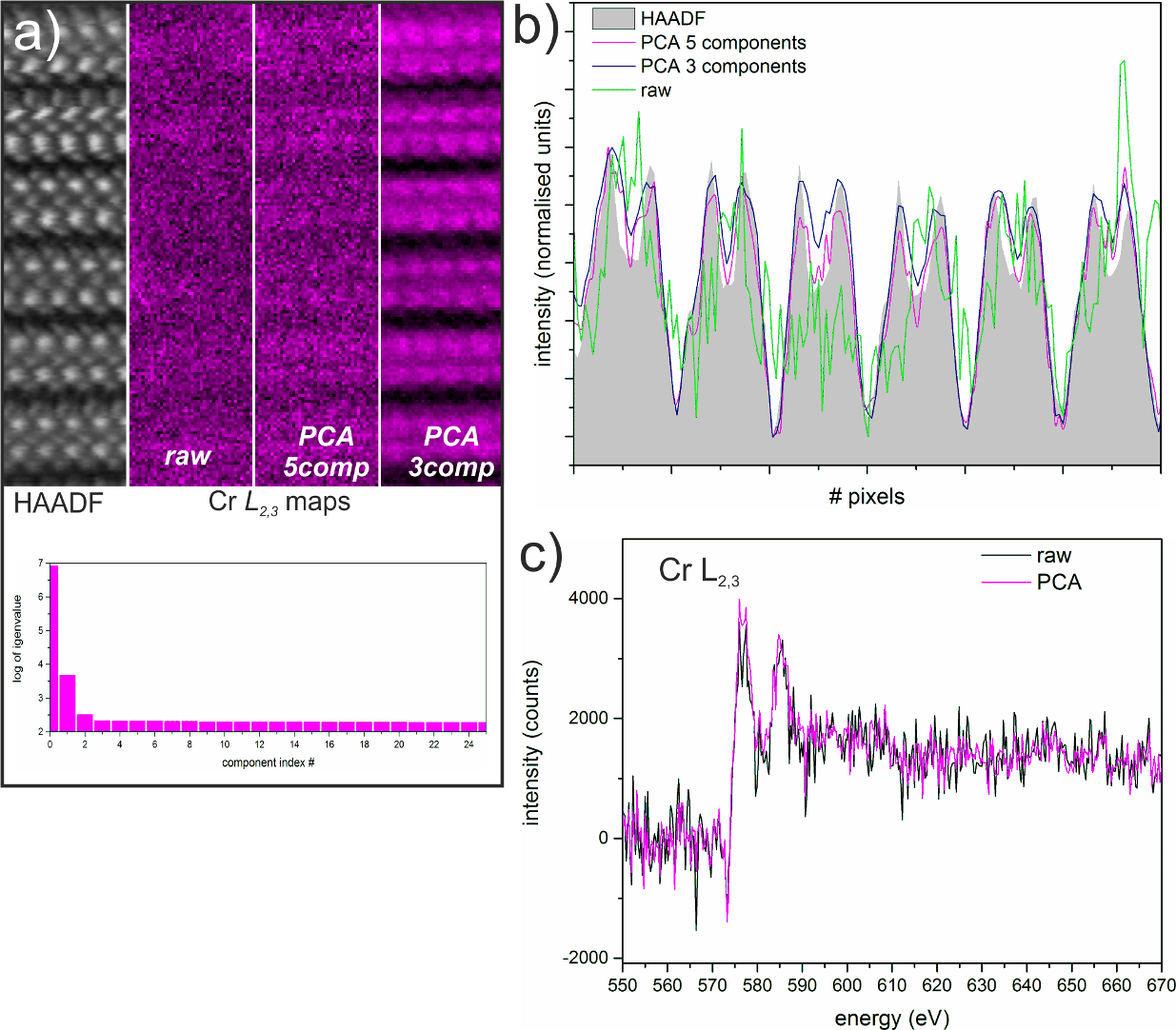


**Figure S4**: a) HAADF STEM signal, Cr *L_2,3_* intensity maps (raw and treated by PCA, respectively) and Scree plot, b) integrated intensity profiles of the HAADF signal and Cr L_2,3_ maps corresponding to a). c) Background subtracted Cr *L_2,3_* EELS edge before and after PCA (3components) showing the reduction of noise (background subtraction performed after PCA).

1. **Principle Component Analysis**

In order to enhance the contrast of EELS chemical maps, data (acquired at an energy dispersion of 0.2eV/channel) were treated by Principle Component Analysis using the CIME plugin for Digital Micrograph. The raw maps are presented alongside the denoised Cr *L_2,3_* maps (Figure S4a); namely data reconstructed using 3 and 5 components. The choice of 3 components was based on the scree plot of the dataset, (Figure S4a, shows that the) which flattens out after component 3, indicating that the remaining factors relate to a very small proportion of the variability and are likely attributed to noise (P. Trebbia, N. Bonnet, Ultramicroscopy 34 (1990) 165-178. Although not atomically resolved the raw data already shows a Cr signal variation, with the higher intensity localized in the QL. Data treated by PCA reveal that the maxima of EELS intensities are localized in the Bi columns. To make this point clear, we have plotted the integrated intensity profiles of the maps (Figure S4b) against the integrated intensity profile of the simultaneously acquired HAADF signal. The intensity profile of the unprocessed data follows the general trend of the HAADF signal, localizing the Cr signal within the quintuple Bi_2_Se_3_ layers. Upon PCA treatment in is evident that the maxima of the Cr *L*_2,3_ intensity profile coincide with the maxima of the HAADF intensity profile (Figure S4c & Figure S5d) ; confirming that the Cr dopants are localized in the Bi sites. In order to exclude any processing artefacts, the background subtracted the Cr *L_2,3_* edges before and after PCA (3 components) are plotted in Figure S4c. Both spectra show a clear Cr *L_2,3_* signal with the treated spectra being denoised.

Similarly data acquired at a dispersion of 1eV/channel, including both Cr and Se *L_2,3_* edges show the anti-correlation relationship between Cr and Se signals, confirming that the Cr atoms reside in Bi sites.

**
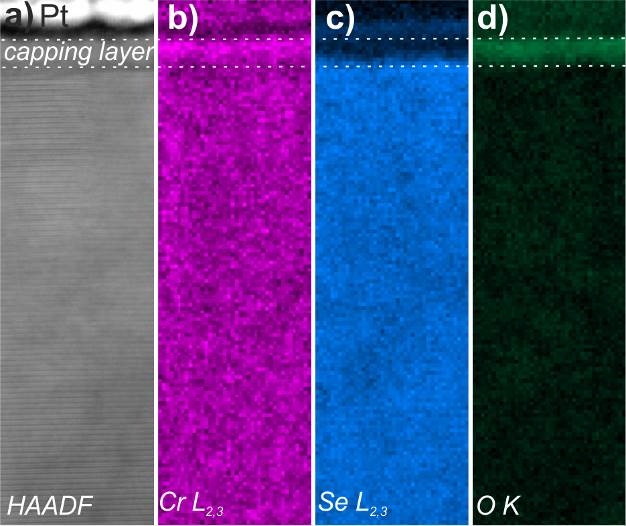
**

**Figure S5**: a) HAADF STEM signal, b,c,d) Cr *L_2r,3_*, Se *L_2,3_* and O *K* intensity maps (data treated by PCA), of the top part of the film showing no Cr segregation at the film surface. Note that the related to a Cr oxide capping layer.

**
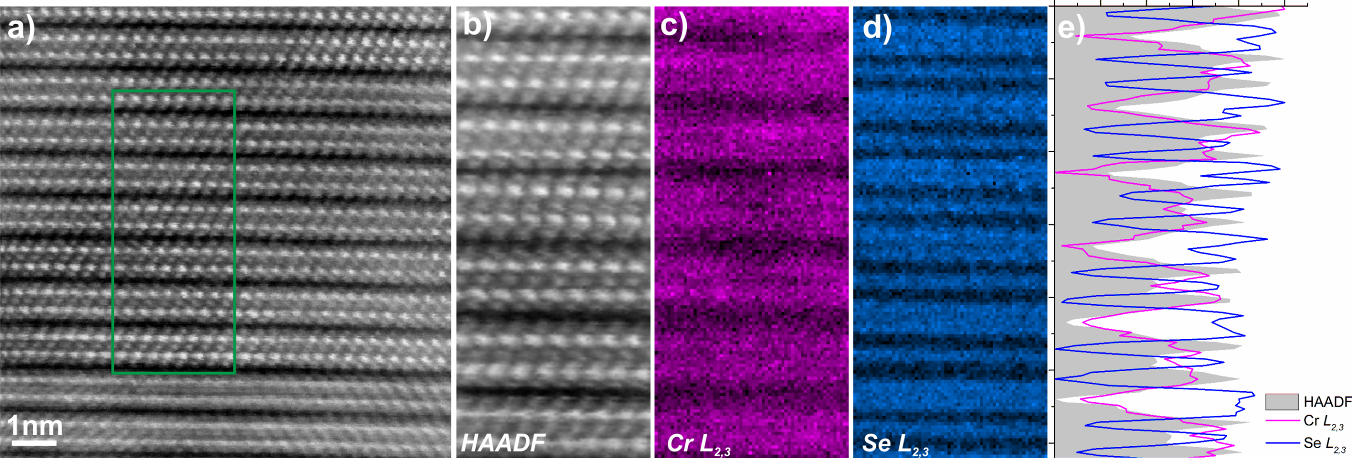
**

**Figure S6**: a) HAADF STEM survey image, b) HAADF STEM signal, c,d) Cr *L_2,3_* and Se *L_2,3_* intensity maps, respectively (treated by PCA) e) integrated intensity profiles of the HAADF signal, *Cr L_2,3_* and Se *L_2,3_* maps showing the anti-correlation of the Cr and Se intensities.


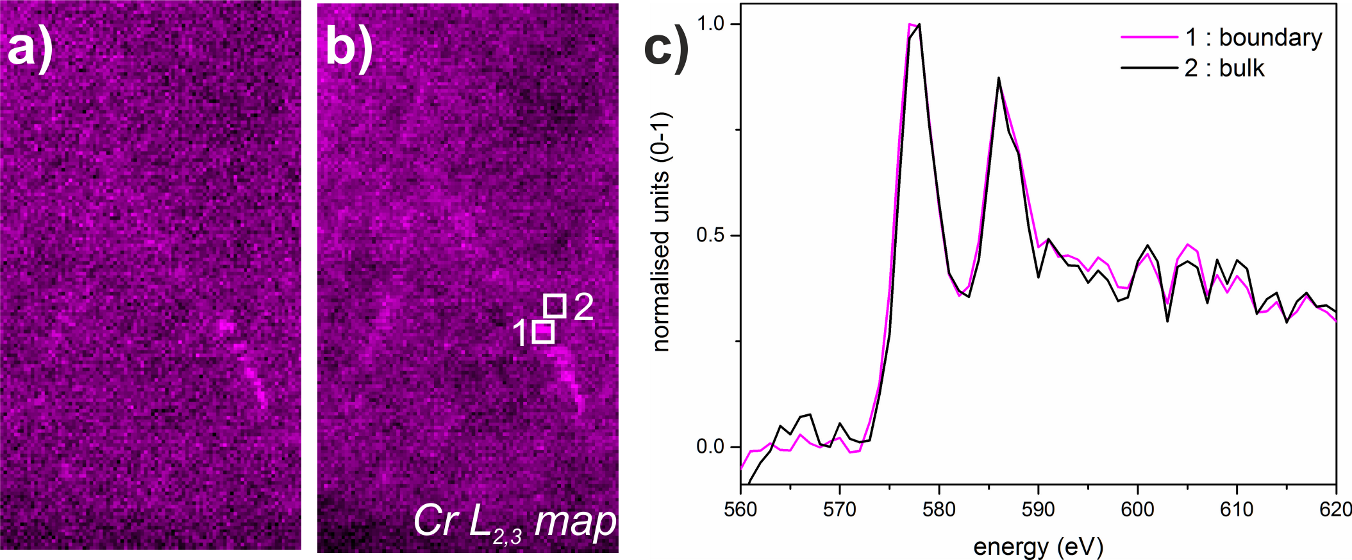


**Figure S6**: EELS maps of the the Cr L_2,3_ edge produced using a) raw and b) PCA treated darta respectively., showing enhanced Cr signal at the grain boundary. The map in b) corresponds to the map showing in **Figure 4c** of the main text. c) background-subtracted Cr L_2,3_ edge extracted from the grain boundary and bulk regions (same as Figure 4e of the main text), normalized to the maximum of the L_3_ peak (background subtraction was performed after PCA).

1. **Discussion on Cr valence state determination by EELS**

Background subtracted Cr L_2,3_ edge extracted from the grain boundary and bulk regions (same as Figure 4e of the main text) were normalized to the maximum of the L_3_ peak. No change can be seen between the two regions, indicating that the Cr in the film is chemically uniform. In both cases the L_3_/L_2_ intensity ratio of Cr was determined to be ~2±0.3.

The determination use of *L*_3_/*L*_2_ intensity ratios is a commonly used method in estimating the oxidation state of transition metals. Recently XAS measurements were used to determine that the valence state of Cr in similar systems is 2^+^ (Liu et al ACS Nano 9(10), 2015, p 023710243, A. I. Figueroa et al., Physical Review B 90 (2014) 134402).

However, the interpretation of this ratio can be ambiguous. The white lines correspond to transitions from 2*p* states to empty *d* states, and hence reflect their occupancy. Consequently, changes in the *L*_3_/*L*_2_ ratios reflect changes in the occupancy of the *d* orbitals, whether those are caused for example by a change in the oxidation state or geometry. Furthermore the resulting near edge fine structure is crucially dependent on several other factors, such as atom coordination, crystal field splitting and spin orbit coupling. Consequently, compounds with the transition metal at the same nominal valence can present different *L*_3_/*L*_2_ ratios. Furthermore the determination of the white line intensity ratio itself can be affected by experimental parameters, for instance convergence and collection semiangles. Data treatment and in particular the choice of background subtraction methodology can yield substantially different results. As a direct consequence, white line intensity ratio values should ideally be compared to others determined under similar experimental and processing parameters, originating from materials systems of similar electronic configurations. A very rigorous and detailed study on the white line ratio of Cr compounds can be found in the paper by T. L. Daulton and B.J. Little, Ultramicroscopy 106 (2006) 561–573; comparing our results to this reference, a nominal valence of 2^+^ could potentially be assigned to Cr in the present samples; however, for the reasons described above this value should only be considered only as indicative.

**References**

S1. P. P. J. Haazen, J. B. Laloe, T. J. Nummy, H. J. M. Swagten, P. Jarillo-Herrero, D. Heiman, and J. S. Moodera, *Appl. Phys. Lett.* **100**, 082404 (2012).
